# Alternative Oxidase Inhibition Impairs Tobacco Root Development and Root Hair Formation

**DOI:** 10.3389/fpls.2021.664792

**Published:** 2021-06-24

**Authors:** Yang Liu, Lu-Lu Yu, Ye Peng, Xin-Xin Geng, Fei Xu

**Affiliations:** ^1^Applied Biotechnology Center, Wuhan University of Bioengineering, Wuhan, China; ^2^Biotechnology Research Center, China Three Gorges University, Yichang, China

**Keywords:** alternative oxidase, auxin, root architecture, respiration, tobacco

## Abstract

Alternative oxidase (AOX) is the terminal oxidase of the mitochondrial respiratory electron transport chain in plant cells and is critical for the balance of mitochondrial hemostasis. In this study, the effect of inhibition of AOX with different concentrations of salicylhydroxamic acid (SHAM) on the tobacco root development was investigated. We show here that AOX inhibition significantly impaired the development of the main root and root hair formation of tobacco. The length of the main root of SHAM-treated tobacco was significantly shorter than that of the control, and no root hairs were formed after treatment with a concentration of 1 mM SHAM or more. The transcriptome analysis showed that AOX inhibition by 1 mM SHAM involved in the regulation of gene expression related to root architecture. A total of 5,855 differentially expressed genes (DEGs) were obtained by comparing SHAM-treated roots with control. Of these, the gene expression related to auxin biosynthesis and perception were significantly downregulated by 1 mM SHAM. Similarly, genes related to cell wall loosening, cell cycle, and root meristem growth factor 1 (RGF1) also showed downregulation on SHAM treatment. Moreover, combined with the results of physiological measurements, the transcriptome analysis demonstrated that AOX inhibition resulted in excessive accumulation of reactive oxygen species in roots, which further induced oxidative damage and cell apoptosis. It is worth noting that when indoleacetic acid (20 nM) and dimethylthiourea (10 mM) were added to the medium containing SHAM, the defects of tobacco root development were alleviated, but to a limited extent. Together, these findings indicated that AOX-mediated respiratory pathway plays a crucial role in the tobacco root development, including root hair formation.

## Introduction

The plant root is crucial for the anchorage of plants in the soil as well as for the uptake of water and nutrients, in addition to sensing and responding to changes in the immediate environment (Tsukagoshi, 2016; Oh et al., 2018). Plant roots can be roughly divided into three regions along their longitudinal axis, namely, a meristematic zone, an elongation zone, and a maturation zone. Among these, the maturation zone is characterized by cells that have completed elongation and are strongly committed to differentiation, including root hair development (Tsukagoshi, 2016).

Root hairs are developed from specific cells of the root epidermis and concentrate on a certain area of the root tip. The number of root hairs of higher plants that develop normally is large, accounting for 77% of the root surface area, and is an important organ for plants to absorb nutrients and water (Parker et al., 2000). There are many factors that are involved in regulating the development of plant root hair. For instance, both the length and the abundance of root hairs are responsive to environmental signals; this ensures the optimal acquisition of soil resources (Salazar-Henao et al., 2016). Moreover, the plant hormones, such as auxin and ethylene, have been demonstrated to play regulatory roles in root hair development (Pitts et al., 1998; Rahman et al., 2002; Vissenberg et al., 2020). However, auxin is a key regulator of root hair formation, which plays as an organizing node for environmental/hormonal pathways to modulate root hair growth (Lee and Cho, 2013). In addition, auxin is also an important modulator of root hair shape and size, and proper auxin distribution is required for correct cell fate assignment, i.e., the determination of initiation site selection during root hair development (Salazar-Henao et al., 2016). Studies with various auxin transporters have demonstrated that the auxin homeostasis is critical for root hair development (Ljung, 2013). Therefore, many earlier studies have shown that auxin-insensitive signaling and transport mutants, such as *axr1*, *axr2*, and *aux1*, show shorter and fewer root hair phenotypes (Pitts et al., 1998; Lee and Cho, 2013).

In addition to phytohormones, reactive oxygen species (ROS) is also an important signaling molecule that affects root hair elongation (Carol and Dolan, 2006). ROS refers to the single-electron reduction product of a class of oxygen produced during the metabolism of organisms, e.g., superoxide anion (O2^.−^), hydrogen peroxide (H_2_O_2_), hydroxyl radical (.OH), etc. (Choudhury et al., 2017). The study found that ROS is an essential substance in the early stage of root hair development. Low concentration of ROS can be used as a second messenger to participate in the development of root hair (Noctor and Foyer, 2016). High concentration of ROS increases the ductility of the cell wall, thereby promoting the root hair polar growth (Takeda et al., 2008). ROS are produced in plant roots by the activity of enzymes located on the plasma membrane, nicotinamide adenine dinucleotide phosphate (NADPH) oxidases that are also called as respiratory burst oxidase homologs (RBOHs) in plant, and by the process of respiration in mitochondria (Suzuki et al., 2011; Tsukagoshi, 2016). It was shown that loss of *Root Hair Defective 2* (*RHD2*), which encodes RBOHC or NADPH oxidase, fails to accumulate ROS at the tip of an incipient root hair and does not develop root hairs (Foreman et al., 2003; Tsukagoshi, 2016). Notably, it has been demonstrated that the amount and the distribution of ROS are crucial for proper root hair morphogenesis.

Alternative oxidase (AOX) is the oxidase at the end of the mitochondrial respiratory electron transport chain, which is widely present in higher plants and plays an important role in the control of ROS accumulation in mitochondria (Fiorani et al., 2005; Xu et al., 2012b). Studies have shown that AOX and its mediated cyanide-resistant respiration pathway are also involved in many physiological and biochemical processes of plants, such as flowering and pollination (Meeuse, 1975), fruit ripening (Xu et al., 2012a; Neelwarne, 2015), resistance to adversity (Selinski et al., 2018; Yu et al., 2021), delaying senescence (Amor et al., 2000), and so on. Interestingly, it was found that inhibiting AOX affected the plant root development of olive (*Olea europaea* L.), especially the development of lateral roots (Santos Macedo et al., 2012). Nevertheless, since then, there have been few reports on the relationship between AOX and root development. In particular, the mechanism of AOX involved in root architecture has not been well understood.

To further explore the influence of AOX inhibition on root development and root hair formation, the model plant tobacco (*Nicotiana tabacum* L.) was used as material in this study. In addition, salicylhydroxamic acid (SHAM), a kind of AOX inhibitor, was applied at different concentrations to investigate the effects of AOX inhibition on the tobacco root architecture, and the changes in gene expression on SHAM treatment were analyzed by RNA-sequencing (RNA-Seq). Our results suggest that AOX plays a crucial role in the tobacco root development and root hair formation. Inhibition of AOX hinders root architecture by promoting ROS excessive accumulation and restraining auxin synthesis and perception.

## Materials and Methods

### Plant Material and Growth Conditions

The seeds of wild-type tobacco (*N. tabacum* L. cv. NC89) were surface-sterilized with 10% NaClO for 15 min and sown on 1/2 Murashige and Skoog (MS) medium containing different levels of effectors. Seeds were germinated and grown in a controlled environment room where day and night temperatures were 25 and 23°C under 16-h-light/8-h-dark photoperiod and 70% relative humanity.

### Chemical Treatments

For SHAM (Tokyo Chemical Industry, Japan) treatments, the seeds were germinated in 1/2 MS containing 1, 2, 3, 4, and 5 mM SHAM, respectively, under the same conditions as mentioned earlier. In addition, methyl jasmonate (MeJA) and auxin [indoleacetic acid (IAA)] were used to study the restoration of tobacco root development while AOX was inhibited. The different concentrations of MeJA and IAA were added to the media as described earlier with some modifications (Zhu et al., 2006; Ivanova et al., 2014). Dimethylthiourea (DMTU), an H_2_O_2_ scavenger, was used at different concentrations according to the earlier methods with some modification (Xia et al., 2009; Chandrakar and Keshavkant, 2018).

### Root Length Measurement

After the germination of tobacco seeds, at least 30 plants under different treatment conditions were taken every day to measure the length of main roots. Then, the development of root hairs was further recorded using a stereomicroscope (SteREODiscovery.V20, Carl Zeiss AG, Germany).

### Reactive Oxygen Species Measurement

The reactive oxygen species (ROS) assay was performed according to the method of Xu et al. (2012b). For superoxide ion measurement, the seedlings were transferred into 0.5 mM Nitrotetrazolium blue chloride (NBT; Sigma, St Louis, MO, United States) solution and stained for 2 h under vacuum filtration in the dark. Seedlings were then decolorized in boiling ethanol (95%) for approximately 20 min. The total intensities of NBT staining in the root part were measured in 15 roots with three biological replicates using the Fiji software package of ImageJ.

The H_2_O_2_ content of seedlings was measured as described by Velikova et al. (2000). Approximately 0.5 g of sample was cut into small pieces and homogenized in an ice bath with 5 ml 0.1% (w/v) trichloroacetic acid. The homogenate was centrifuged at 12,000 × *g* for 20 min at 4°C, and then 0.5 ml of the supernatant was added to 0.5 ml of 10 mM potassium phosphate buffer (pH 7.0) and 1 ml of 1 M KI. The absorbance of the supernatant was read at 390 nm.

### Cell Death Determination

The dead cells on SHAM treatments were visually detected using a trypan blue staining method as described by Han et al. (2017) with some modifications. Tobacco roots were stained with lactophenol–trypan blue solution (i.e., 10 ml of lactic acid, 10 ml of glycerol, 10 g of phenol, and 10 mg of trypan blue, dissolved in 10 ml of distilled water) at boiled water for 1 min and then left staining at room temperature for another 10 min. Then, samples were placed in chloral hydrate solution (i.e., 2.5 g of chloral hydrate dissolved in 1 ml of distilled water) to reduce background staining and then were equilibrated with 70% glycerol for scanning. To observe the root cell death, samples were stained with 10 μg/ml of propidium iodide (PI) for 5 min and then rinsed with ddH_2_O. Fluoresce images were obtained using a Leica microscope (EVOS FL Imaging System, Life Technologies Corp., Washington, United States).

### Respiration Measurements

The respiration measurement was performed according to the method of Yu et al. (2020). Approximately 50 mg of root tissues were cut into small pieces, and then transferred into air-tight cuvettes containing 20 mM 2-[4-(2-hydroxyethyl)piperazin-1-yl]ethanesulfonic acid (HEPES) and 0.2 mM CaCl_2_ with pH 7.2, and oxygen uptake was measured as a decrease of O_2_ concentration in the dark using a Clark-type oxygen electrode (Chlorolab2, Hansatech, United Kingdom). Total respiration rate (*V*_t_) was measured without any inhibitors. Then, 1 mM KCN was added to inhibit cytochrome *c* pathway respiration (*V*_cyt_), which represents the sum of the AOX alternative pathway respiration rate (*V*_alt_) and the residual respiration rate (*V*_res_). Then, 1 mM *n*-propyl gallate (nPG) was added to inhibit the *V*_alt_ and got the *V*_res_ value. Therefore, the *V*_alt_ was defined as O_2_ uptake rate without *V*_cyt_ and *V*_res_.

### RNA Extraction and Transcriptome Sequencing

For the RNA-Seq analysis, tobacco root tissues on the 8th day after treatment with or without 1 mM SHAM were used in this study. The subsequent experiments of tobacco root tissues include the extraction, purification, analysis, and sequencing of total RNA by Novogene Bioinformatics Technology Co. Ltd. (Beijing, China). Sequencing libraries were generated using NEBNext® Ultra™ RNA Library Prep Kit for Illumina® (NEB, Ipswich, MA, United States) according to the instructions of the manufacturer, and index codes were added to attribute sequences to each sample.

### Quantification of Gene Expression Levels

HISAT2 was used to count the number of reads mapped to each gene. In addition, the fragments per kilobase of exon model per million mapped reads (FPKM) of each gene were calculated based on the length of the gene and the number of reads mapped to the gene.

### Differential Expression Analysis

The differential expression analysis was performed using the DESeq2 R package (version 1.16.1). DESeq2 provide statistical routines for determining the differential expression in the digital gene expression data using a model based on the negative binomial distribution. The resulting value of *p* were adjusted using the Benjamini–Hochberg approach for controlling the false discovery rate. Genes with an adjusted *p* < 0.05 found by DESeq2 were assigned as differentially expressed.

### Gene Ontology and KEGG Enrichment Analysis

The Gene Ontology (GO) enrichment analysis of differentially expressed genes (DEGs) was implemented by the cluster Profiler R package, in which gene length bias was corrected. GO terms with corrected value of *p* < 0.05 were considered significantly enriched by DEGs.

The KEGG is a database resource for understanding the high-level functions and utilities of the biological system, such as the cell, the organism, and the ecosystem, from molecular-level information, especially large-scale molecular data sets generated by genome sequencing and other high-throughput experimental technologies.^1^ We used the cluster Profiler R package to test the statistical enrichment of DEGs in KEGG pathways.

### Real-Time Quantitative PCR Analysis

In order to validate the results from the transcriptome sequencing analysis, a part of genes was confirmed by the real-time quantitative PCR (qRT-PCR), and *Actin* (Accession number: AB158612) gene was used as an internal control. All the primers are listed in Supplementary Table S1. The qRT-PCRs were prepared with the SYBR Green Master Mix Reagent (Applied Biosystems, Waltham, MA, United States), following the instructions of the manufacturer. The reactions were carried out in Applied Real-Time System (ABI 7500). All samples were performed in triplicate, and the relative expression levels were calculated using the 2^−ΔΔCT^ method of the system.

### Statistical Analysis

The statistical analysis of the results from three independent experiments with nine measurements used a one-way ANOVA, followed by Tukey’s HSD *post hoc* test or Dunnett’s HSD test. Asterisks or different letters in graphs indicate the level of significance (*p* < 0.05).

## Results

### Inhibition of AOX Impairs the Root Development of Tobacco

**Figure 1:**
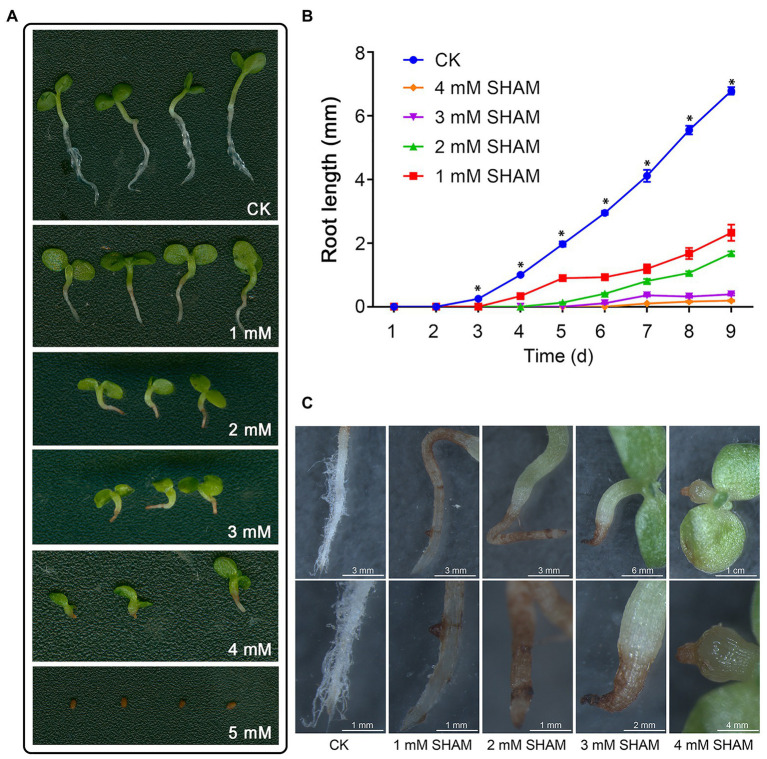
Effects of salicylhydroxamic acid (SHAM) treatment on root formation of tobacco. **(A)** Comparison of tobacco root development under normal conditions (CK) and different concentrations of SHAM. **(B)** Comparison of root length in the absence (CK) and presence of SHAM treatments. The data are the means ± SD of three independent experiments from at least 30 seedlings. The significant differences between the SHAM-treated samples and the control samples are denoted by asterisks, ^∗^*p* < 0.05. **(C)** Microscopic comparison of root phenotypes in the absence (CK) and presence of SHAM treatments. Seedlings on 8th day of growth were used for observation and comparison.

To investigate the effects of AOX inhibition on the tobacco root development, different concentrations of SHAM were applied and the root growth was compared (Figure 1; Supplementary Figure S1). As shown in Figure 1, the root development including main roots and root hairs of tobacco was greatly inhibited by SHAM treatments compared with control. Under the conditions of 1 and 2 mM SHAM, the root hair development was markedly blocked and the length of the main root was obviously shorter than the control (Figure 1A). When the higher concentrations of SHAM were applied, the development of tobacco root systems was further inhibited, especially under the condition of 5 mM SHAM (Figures 1A,B). The microscopic observations revealed that the SHAM-treated tobacco showed lacking of root hair around the root system, and the main roots of tobacco showed wilting and browning, especially at the tip part (Figure 1C). Moreover, it should be noted that when the concentration of SHAM treatment reached 4 mM, the root development was completely blocked (Figure 1A).

### Inhibition of AOX Suppresses Cellular Respiration and Promotes ROS Accumulation

**Figure 2:**
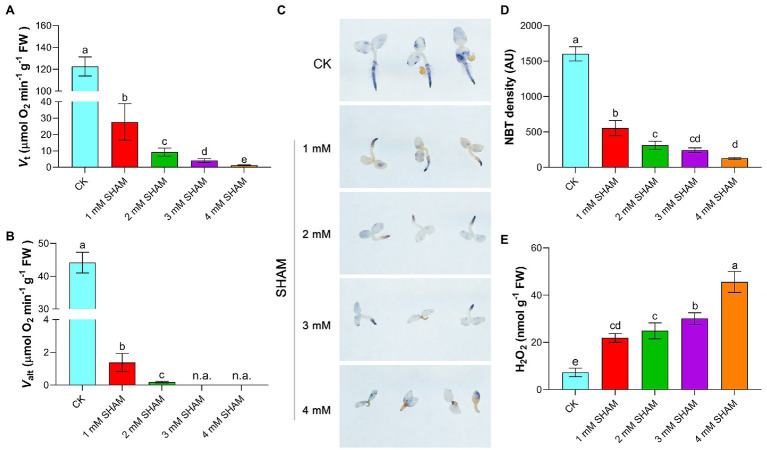
Comparison of oxidative damage and respiration rate between the SHAM-treated seedlings and the control (CK). **(A,B)** Changes of total respiration (*V*_t_) and alternative pathway respiration (*V*_alt_) in the absence (CK) or presence of SHAM treatments. **(C)** Determination of superoxide ion with 0.5 mM nitrotetrazolium blue chloride (NBT) for 2 h. **(D)** Quantification of NBT staining intensity in the main root zone using ImageJ software; AU, arbitrary units. **(E)** Comparison of H_2_O_2_ content between SHAM-treated seedlings and the control (CK). Seedlings on 8th day of growth were used for determination. The data are the means ± SD of three independent experiments from at least 30 seedlings. The significant differences are denoted by different letters. n.a., not available.

Considering that AOX mediates branch respiration in the mitochondrial electron transport chain, the total respiration (*V*_t_) and alternative pathway respiration (*V*_alt_) of root were determined under different concentrations of SHAM. As shown in Figure 2A, SHAM treatments suppressed the *V*_t_ and *V*_alt_ of tobacco roots and this inhibition became more obvious with the increase of SHAM concentration. Under the conditions of 1 mM SHAM, the *V*_t_ of tobacco roots was four times lower than that under the normal conditions (Figure 2A). When 2 mM SHAM was applied, the *V*_t_ decreased to 10 times lower than that of the control. Certainly, more serious respiration inhibition happened when seedlings suffered from 3 to 4 mM SHAM treatment (Figure 2A). Similarly, the *V*_alt_ of tobacco roots was significantly suppressed by SHAM treatments, especially under the conditions of 3 and 4 mM SHAM, where the *V*_alt_ of tobacco roots was almost undetectable (Figure 2B).

It was observed that, moreover, there was a large amount of O2^.−^ accumulation in the root system during tobacco root development under normal growth conditions, especially at the root tip (Figure 2C). In contrary, tobacco seedlings grown under different concentrations of SHAM showed less O2^.−^ content than the control, especially under the higher concentrations of SHAM conditions such as 4 mM SHAM. When the higher concentration of SHAM was applied, the lower level of O2^.−^ was detected in the root (Figures 2C,D). However, the H_2_O_2_ assay showed that there were more H_2_O_2_ contents accumulated in SHAM-treated seedlings than that in the control seedlings (Figure 2E).

### Inhibition of AOX Causes Root Cell Death

**Figure 3:**
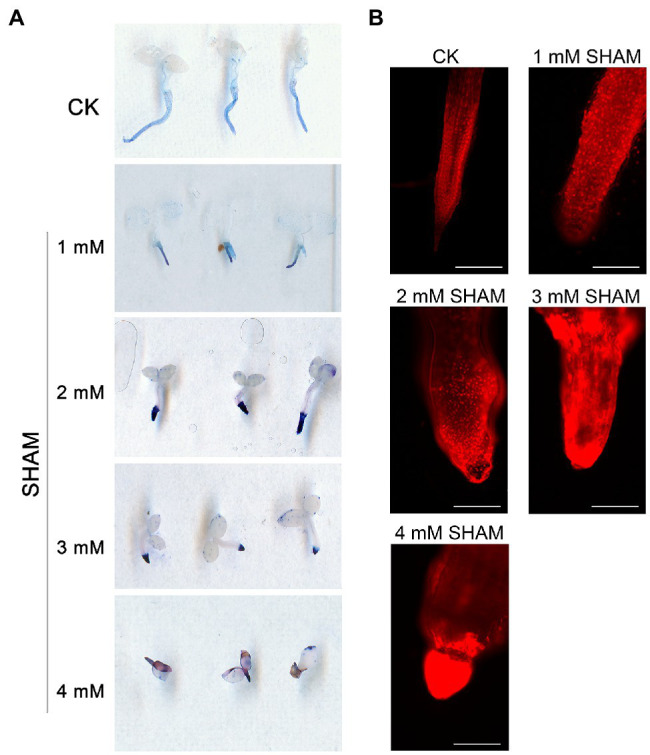
Comparison of cell death between the SHAM-treated seedlings and the control (CK). **(A)** Cell death was determined with trypan blue, and the representative roots are shown. **(B)** The microscopic images of roots after propidium iodide (PI) staining and the representative roots are shown. Seedlings on 8th day of growth were used for trypan blue and PI staining. Scale bar, 200 μm.

In addition to ROS burst, it was worth noting that the inhibition of AOX with different concentrations of SHAM caused an obvious cell death in the root system based on the trypan blue assay, and this effect became more serious as higher concentrations of SHAM were applied (Figure 3). Results showed that tobacco roots were stained light blue by trypan blue under normal culture condition, whereas the color became more obvious under SHAM treatments (Figure 3A). In addition, the microscopic observation of the roots after PI treatment showed that the root cell damage was the same as the results of trypan blue staining. When compared with control, the cell death of tobacco roots under the SHAM treatment conditions was more obvious, especially the part embedded in the medium (Figure 3B). These results together with the ROS analysis implied that AOX inhibition led to an excessive increase in ROS, which then caused cell death in the root system.

### Identification of Differentially Expressed Genes Affected by AOX Inhibition

**Figure 4:**
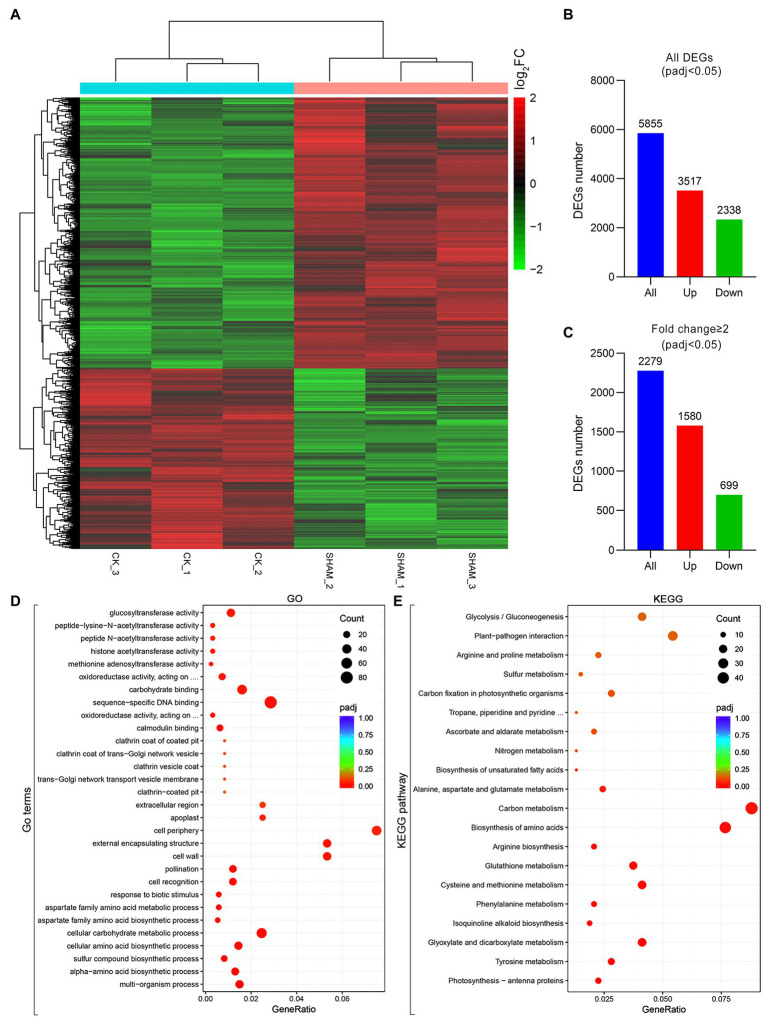
Overview of changes in gene expression profiles of tobacco on SHAM treatment. **(A)** Heat map diagrams showed the relative expression levels of total differentially expressed genes (DEGs). **(B)** Number of DEGs includes up- and downregulated genes on SHAM treatment, *p*_adj_ < 0.05. **(C)** Number of DEGs (fold change ≥2) in SAHM vs. CK. log_2_FC, log_2_FoldChange. **(D)** Gene Ontology (GO) terms enriched in SHAM vs. CK. **(E)** KEGG pathway enriched in SHAM vs. CK.

Given that both the main roots and root hairs were obviously inhibited by 1 mM SHAM, this concentration was used in the RNA-Seq analysis to reveal the regulation of gene expression affected by AOX inhibition (Figure 4A). As shown in Figure 4, a total of 5,855 DEGs were identified, of which 3,517 were upregulated genes and 2,338 were downregulated genes (Figure 4B). If fold change ≥2 was considered as a basic threshold, 2,279 DEGs were detected, of which 1,580 were upregulated and 699 were downregulated by SHAM treatment (Figure 4C). To confirm the reliability of the transcriptome sequencing, parts of DEGs were investigated by qRT-PCR. As shown in Supplementary Figure S2, the results of qRT-PCR were generally consistent with the transcriptome data, suggesting a strong positive correlation between the qRT-PCR and transcriptome data.

**Figure 5:**
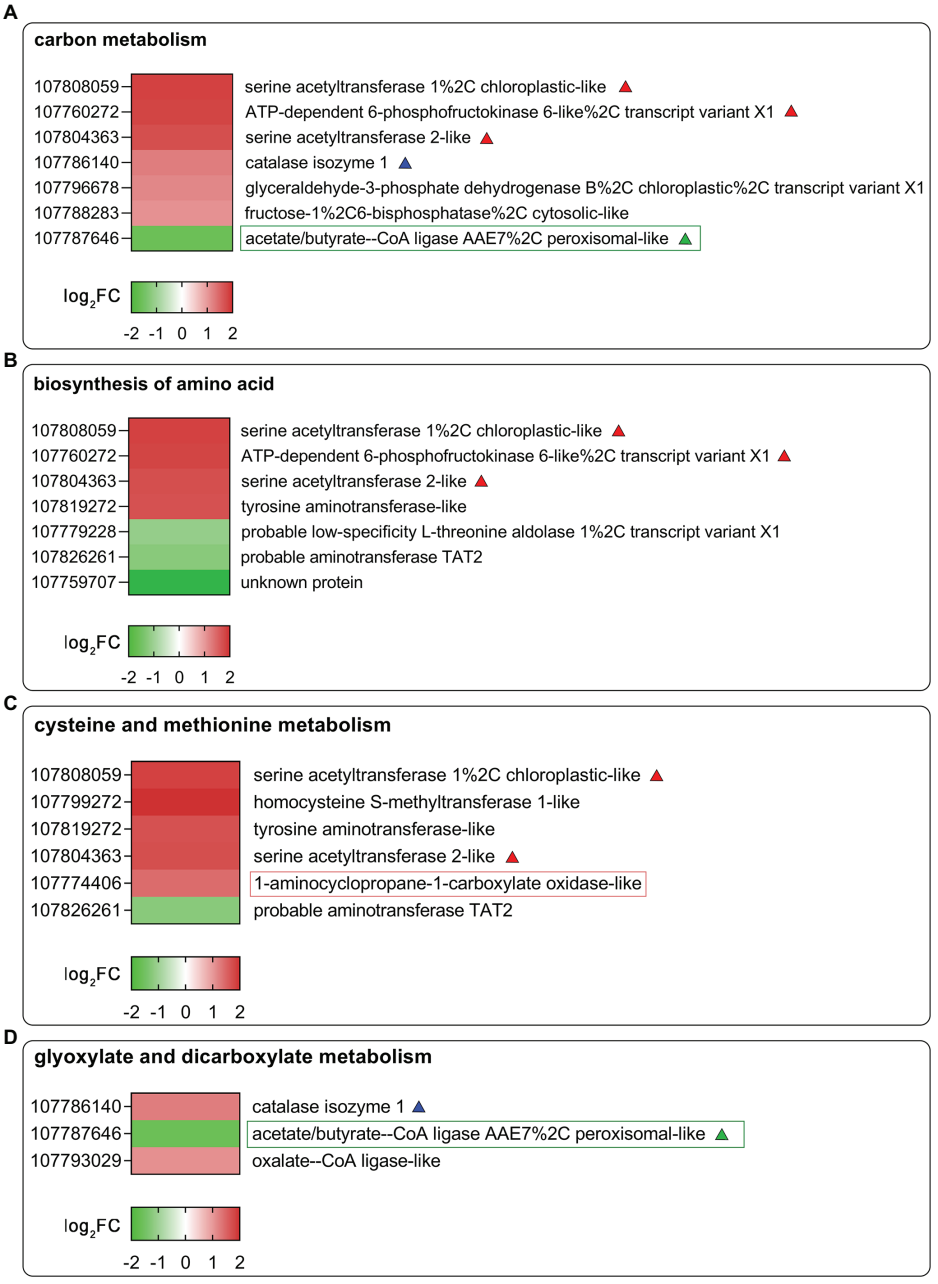
Heat map diagrams of DEGs related to carbon metabolism **(A)**, biosynthesis of amino acids **(B)**, glyoxylate and dicarboxylate metabolism **(C)**, and cysteine and methionine metabolism **(D)** in response to SHAM treatment. DEGs of |log_2_FC ≥ 1| are shown in the image. log_2_FC, log_2_FoldChange. The red-, blue-, and green-colored triangles or rectangles represent DEGs that appear in different KEGG pathways.

To further identify the functions of DEGs regulated by SHAM treatment, the GO and KEGG analyses were then performed. As shown in Figure 4D, the DEGs of SHAM vs. control (CK) were enriched in GO terms of the cell wall, cell periphery, and external encapsulating structure, etc. The KEGG pathway analysis showed that most of DEGs were markedly enriched in “Carbon metabolism”, “Biosynthesis of amino acids”, “Glyoxylate and dicarboxylate metabolism”, and “Cysteine and methionine metabolism” (Figure 4E). It should be noted that several DEGs (|log_2_FC ≥ 1|) were commonly enriched in these pathways, i.e., LOC107808059 and LOC107804363, which encode serine acetyltransferase, were enriched in the KEGG pathways of “Carbon metabolism”, “Biosynthesis of amino acids”, and “Cysteine and methionine metabolism”, and showed upregulation on SHAM treatment (Figures 5A–C). In addition, the gene expression of LOC107826261, which encodes aminotransferase and belongs to KEGG pathways of “Biosynthesis of amino acids” and “Cysteine and methionine metabolism”, was downregulated by SHAM treatment (Figures 5B,C). Similarly, the gene expression of LOC107787646, which encodes acetate/butyrate-CoA ligase, was also significantly downregulated by SHAM treatment (Figures 5A,D). Notably, LOC107774406, which encodes 1-aminocyclopropane-1-carboxylate oxidase and is a key enzyme of ethylene biosynthesis pathway, showed the upregulated gene expression on SHAM treatment. These findings indicate that AOX inhibition affects the anabolic and catabolic processes directly or indirectly.

### Expression Profiling of Genes Related to ROS Scavenging System

**Figure 6:**
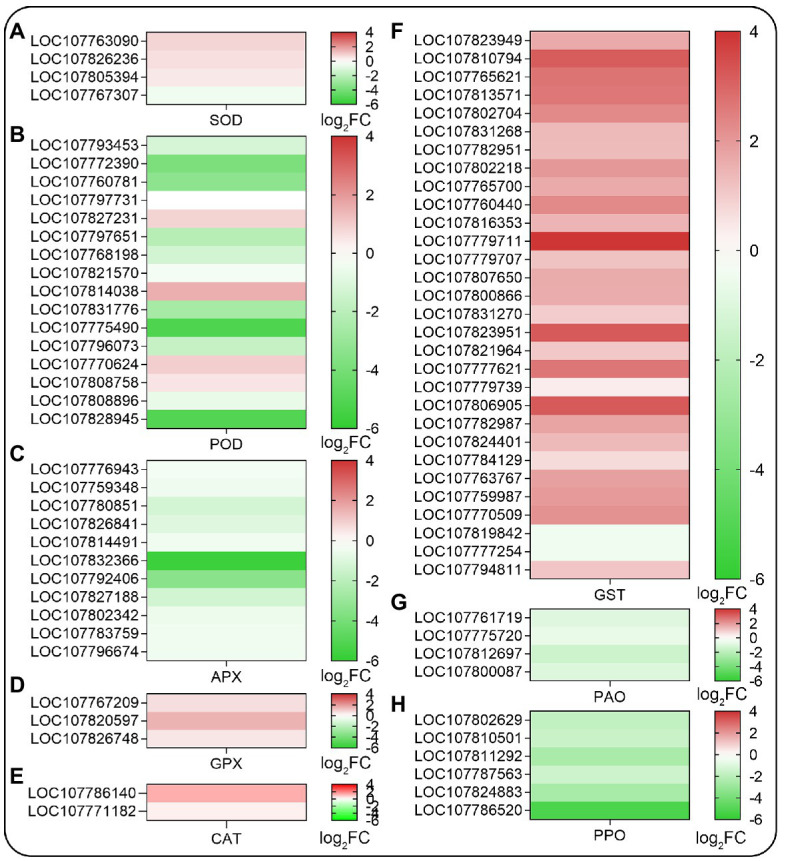
Heat map diagrams of relative expression levels of reactive oxygen species (ROS) scavenging-related genes in response to SHAM treatment. **(A)** SOD, superoxide dismutase; **(B)** POD, peroxidase; **(C)** APX, ascorbate peroxidase; **(D)** GPX, glutathione peroxidase; **(E)** CAT, catalase; **(F)** GST, glutathione *S*-transferase; **(G)** PAO, polyamine oxidase; and **(H)** PPO, polyphenol oxidase. log_2_FC, log_2_FoldChange.

Since SHAM treatment affected the accumulation of ROS in roots, then the ROS-related genes were analyzed further. As shown in Figure 6, the genes related to antioxidant systems such as superoxide dismutase (SOD), peroxidase (POD), ascorbate peroxidase (APX), glutathione peroxidase (GPX), and glutathione *S*-transferase (GST) were affected by AOX inhibition. Among these, the expression of a small amount of DEGs related to SOD and GPX were upregulated, but a large amount of DEGs related to POD and APX were downregulated by SHAM (Figures 6A–E). It should be noted that the DEGs related to GST, which plays a key role in glutathione binding reaction, and their expressions were significantly upregulated by SHAM treatment (Figure 6F). Notably, further analysis showed that the DEGs related to polyphenol oxidase (PPO) and polyamine oxidase (PAO) exhibited downregulation after SHAM treatment (Figures 6G,H).

### Expression Profiling of Genes Related to Auxin Biosynthesis and Signaling Transduction Pathway

**Figure 7:**
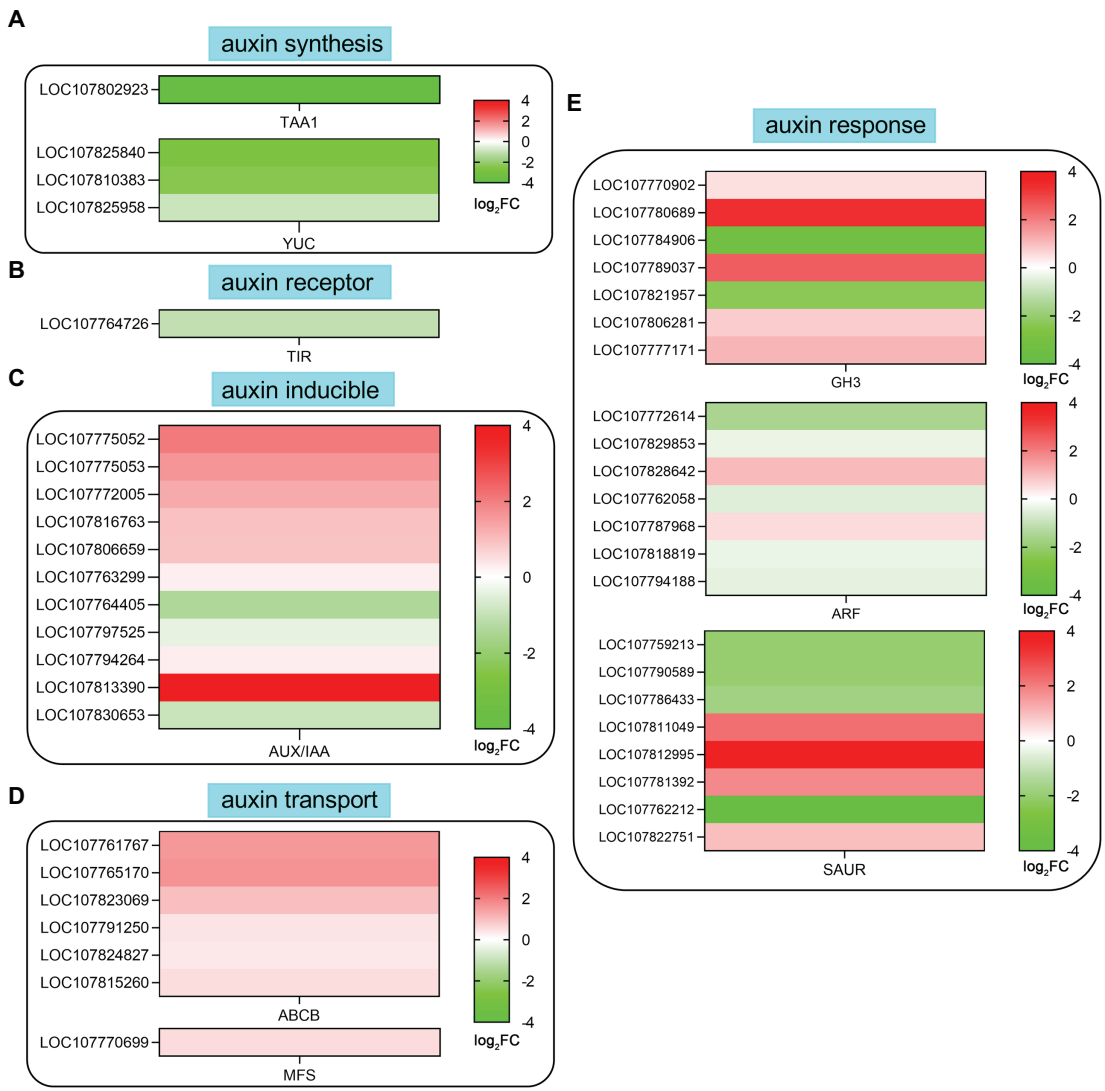
Effects of SHAM treatment on the gene expression of auxin biosynthesis and signaling transduction pathway. DEGs related to auxin biosynthesis **(A)**, auxin receptor **(B)**, auxin inducible **(C)**, auxin transport **(D)**, and auxin response **(E)** are shown. TAA1, tryptophan aminotransferase; TIR1, transport inhibitor response 1; ABP, auxin binding protein; SAUR, small auxin-up RNA; ABCB, ABC transporter B family member; MFS, multidrug resistance protein; GH3, indole-3-acetic acid-amido synthetase GH3; and ARF, auxin response factor. log_2_FC, log_2_FoldChange.

Many studies have shown that auxin is the main signaling molecule involved in regulating root development and root hair growth. In this study, auxin pathway-related genes were downregulated or upregulated by SHAM treatment, such as tryptophan aminotransferase (*TAA*), flavin-containing monooxygenases (*YUC*s), and transport inhibitor response 1 (*TIR1*; Figure 7). As shown in Figure 7A, one *TAA* (LOC107802923) and three *YUC*s (LOC107825840, LOC107810383, and LOC107825958) genes were involved in auxin synthesis, and their expressions were significantly downregulated by SHAM treatment. In addition, the expression of *TIR1* (LOC107764726), a gene of auxin receptor, was also downregulated by SHAM treatment (Figure 7B). Moreover, it should be noted that six auxin binding proteins (ABPs), which are also believed to function as an IAA receptor, showed downregulation on SHAM treatment (Figure 7B).

Further analysis showed that the expression of 11 *AUX*/*IAA* genes was affected by SHAM treatment, and most of these genes were upregulated (Figure 7C). Likewise, the DEGs related to auxin transport (efflux carrier) such as ABC transporter B family member (ABCB) and multidrug resistance protein (MFS) showed upregulation on SAHM (Figure 7D). Moreover, many genes involved in auxin response were also regulated by SHAM treatment (Figure 7E). Of these, five auxin response factor (*ARF*) genes showed significant downregulation after SHAM treatment. Besides, seven genes of Gretchen Hagen 3 (GH3) belonging to indole-3-acetic acid-amido synthetase were identified, and the expression of five *GH3* genes was significantly upregulated by SHAM treatment (Figure 7E). In comparison, the gene expressions of small auxin-up RNA (SAUR) were up- and downregulated on SHAM treatment (Figure 7E). These results indicate that AOX inhibition affected the tobacco root formation by suppressing auxin synthesis and signaling transduction.

### Expression Profiling of Genes Related to Ethylene Biosynthesis and Signaling Transduction Pathway

**Figure 8:**
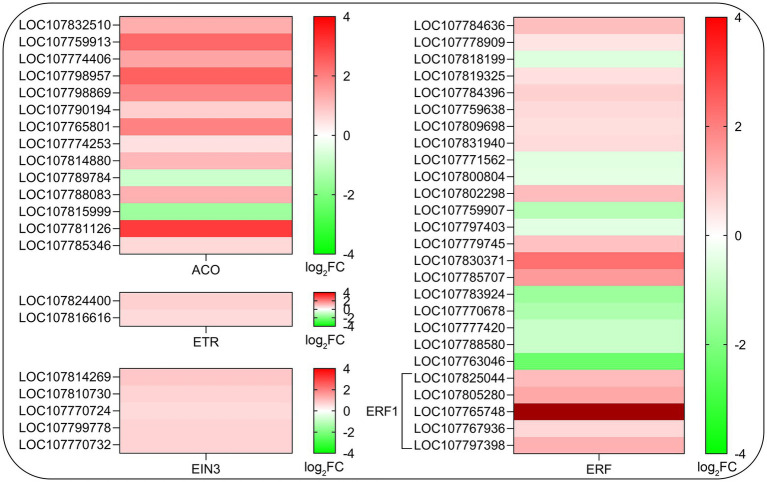
Comparison of DEGs related to ethylene biosynthesis and signaling transduction pathway on SHAM treatment. ACO, 1-aminocyclopropane-1-carboxylate oxidase; ETR, ethylene receptor; EIN3, ethylene insensitive 3; and ERF, ethylene response factor. log_2_FC, log_2_FoldChange.

Considering that ethylene is a positive regulator of root hair development (Tanimoto et al., 1995; Pitts et al., 1998; Lee and Cho, 2013) and some of the key genes related to ethylene biosynthesis were assigned to KEGG pathway (sly00270, *p* < 0.05), we further analyze the effects of SHAM treatment on the gene expression of ethylene biosynthesis and signaling transduction pathway. As shown in Figure 8, a total of 14 DEGs involved in key steps of ethylene biosynthesis such as 1-aminocyclopropane-1-carboxylate oxidase (ACO) were identified and 12 of them showed significant upregulation on SHAM treatment (Figure 8). Moreover, the expression of two genes of ethylene receptor (ETR), five genes of ethylene insensitive 3 (EIN3), and more than 20 genes of ethylene-responsive transcription factor (ERF) were affected differentially by SHAM treatment. Of these, the expressions of *EIN3* genes and *EFR1* genes were upregulated by SHAM treatment (Figure 8). These results suggest that AOX inhibition-mediated defects in root development did not occur through impaired ethylene biosynthesis and signal transduction.

### Expression Profiling of Genes Related to Cell Wall and Root Meristem System

During root development, especially the root hair initiation, the local cell wall loosening is required (Tominaga-Wada et al., 2011). In addition, it has been demonstrated that root meristem size is controlled by root meristem growth factor 1 (RGF1), which affects the root architecture (Yamada et al., 2020). Interestingly, in this study, we found a total of 25 DEGs related to the cell wall, including the genes involved in xyloglucan endotransglucosylase/hydrolase protein and pectinesterase, showed significant downregulation on SHAM treatment (Table 1). Conversely, the gene expressions of pectinesterase inhibitor were upregulated by SHAM treatment (Table 1). It is of interest to note that, moreover, the expression of two *RGF1* genes (LOC107788696, log_2_FC = −7.15; LOC107823248, log_2_FC = −2.84) were significantly downregulated by SHAM treatment (Table 1). These results indicate that AOX-mediated root development defects were involved in the cell wall loosening and root tip meristem formation.

**Table 1:** Differentially expressed genes (DEGs) related to cell wall metabolism.

Gene ID	log_2_FC	*p*	Gene description
107,800,334	−0.87	3.74E-17	Xyloglucan endotransglucosylase/hydrolase protein 9-like
107,787,957	−0.58	1.17E-08	Xyloglucan endotransglucosylase/hydrolase protein 9-like
107,778,234	−0.99	3.03E-16	Xyloglucan endotransglucosylase/hydrolase 2-like
107,789,124	−3.19	7.92E-07	Xyloglucan endotransglucosylase/hydrolase 2-like
107,808,205	−5.43	1.23E-04	Probable xyloglucan endotransglucosylase/hydrolase protein B
107,807,679	−2.91	2.68E-03	Probable xyloglucan endotransglucosylase/hydrolase protein B
107,783,320	−0.57	1.48E-03	Probable xyloglucan endotransglucosylase/hydrolase protein 8
107,791,643	−0.64	3.64E-03	Probable xyloglucan endotransglucosylase/hydrolase protein 8
107,794,176	−3.16	4.82E-06	Probable xyloglucan endotransglucosylase/hydrolase protein 7
107,825,008	−2.10	6.00E-04	Probable xyloglucan endotransglucosylase/hydrolase protein 7
107,805,355	−0.55	3.09E-03	Probable xyloglucan endotransglucosylase/hydrolase protein 32
107,809,788	−0.82	6.76E-09	Probable xyloglucan endotransglucosylase/hydrolase protein 28
107,805,952	−0.66	3.93E-05	Probable xyloglucan endotransglucosylase/hydrolase protein 28
107,805,693	−3.49	9.48E-04	Probable xyloglucan endotransglucosylase/hydrolase protein 26
107,763,397	2.16	3.69E-08	Probable xyloglucan endotransglucosylase/hydrolase protein 30
107,810,365	−5.81	1.52E-04	Probable pectinesterase/pectinesterase inhibitor 46
107,829,076	−4.33	1.01E-03	Probable pectinesterase/pectinesterase inhibitor 46
107,787,878	−1.00	1.21E-06	Probable pectinesterase/pectinesterase inhibitor 25
107,818,643	−0.75	2.63E-06	Probable pectinesterase/pectinesterase inhibitor 25
107,777,616	−0.73	5.05E-08	Probable pectinesterase 53
107,785,386	−1.14	1.31E-04	Pectinesterase-like%2C transcript variant X2
107,791,975	−1.49	7.76E-09	Pectinesterase-like
107,819,367	−0.82	3.72E-03	Pectinesterase-like
107,777,282	−0.52	3.92E-03	Pectinesterase-like
107,766,157	−1.85	1.05E-03	Pectinesterase/pectinesterase inhibitor 18-like
107,770,142	−5.02	1.11E-06	Pectinesterase 2-like
107,775,110	2.55	5.78E-06	Pectinesterase-like
107,832,646	0.80	2.40E-04	Probable pectinesterase/pectinesterase inhibitor 12
107,786,066	1.21	2.50E-07	Probable pectinesterase/pectinesterase inhibitor 12
107,815,259	0.48	3.66E-07	Pectinesterase/pectinesterase inhibitor U1-like
107,811,022	1.07	3.13E-05	Probable pectinesterase/pectinesterase inhibitor 51
107,832,120	1.42	4.69E-03	Probable pectinesterase/pectinesterase inhibitor 40

log_2_FC, log_2_FoldChange.

### Expression Profiling of Genes Related to Cell Cycle

Considering that the root development and root hair formation are related to cell proliferation, we also tried to analyze the effect of SHAM treatment on the expression of cell cycle-related genes. As shown in Table 2, some DEGs related to cyclin-dependent kinase inhibitor protein (CDI), cell division cycle, and cell cycle regulatory genes were identified. Of these, all five genes of CDIs, whose proteins function as constraining the activities of cyclin-dependent kinases (CDKs), showed a significant upregulation under the condition of SHAM treatment (Table 2). In contrast, the expressions of cell division cycle (e.g., LOC107832361, log_2_FC = −2.42) and cell cycle regulatory genes including cyclin D3-1 (*CYCD3;1*, LOC107776168) and cyclin D3-2 (*CYCD3;2*, LOC107792202) were downregulated by SHAM treatment (Table 2).

**Table 2:** DEGs related to cell cycle.

Gene ID	log_2_FC	*p*	Gene description
107,820,152	0.98	4.55E-03	Cyclin-dependent protein kinase inhibitor SMR2-like
107,820,952	0.98	2.56E-05	Cyclin-dependent kinase inhibitor 7-like%2C transcript variant X1
107,815,834	1.37	5.14E-05	Cyclin-dependent kinase inhibitor 7-like
107,830,774	0.46	5.30E-03	Cyclin-dependent kinase inhibitor 5-like
107,810,968	1.05	1.57E-07	Cyclin-dependent kinase inhibitor 1-like
107,792,202	−0.56	2.64E-07	Cyclin-D3-2-like
107,776,168	−0.44	1.94E-03	Cyclin-D3-1-like
107,761,354	0.48	3.99E-03	Cell division cycle protein 48 homolog%2C transcript variant X2
107,832,361	−2.42	2.37E-03	Cell division cycle protein 27 homolog B-like
107,802,770	−0.37	1.11E-03	Cell division cycle and apoptosis regulator protein 1-like
107,807,000	−0.35	4.29E-03	Cell division cycle and apoptosis regulator protein 1-like

log_2_FC, log_2_FoldChange.

### Expression Profiling of Genes Related to Cell Apoptosis

**Figure 9:**
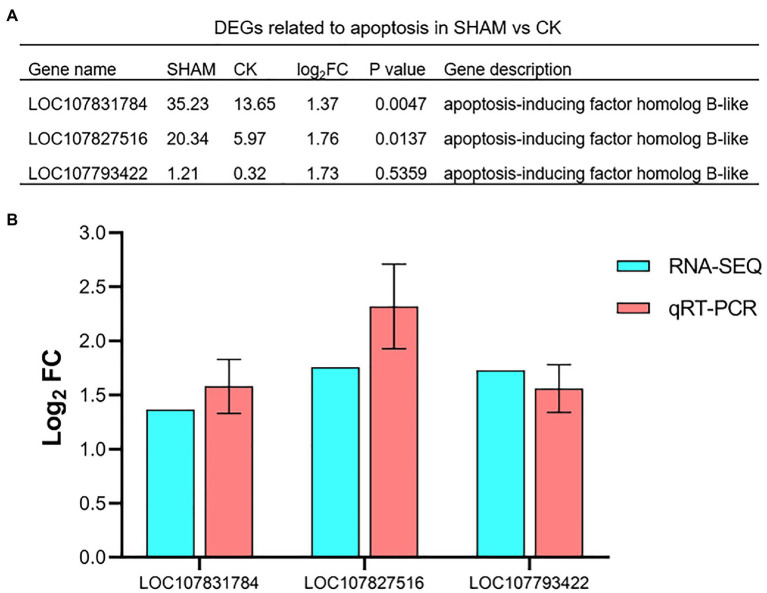
Relative expression levels of apoptosis-related genes in response to SHAM treatment. DEGs related to apoptosis (A) and their quantitative analysis by qRT-PCR. (B). log_2_FC, log_2_FoldChange.

Since the cell apoptosis of the root was clearly observed on SHAM treatment, the DEGs involved in apoptosis were further analyzed. As shown in Figure 9A, three genes of apoptosis-inducing factors were identified. Of these, it is of interest to note that the gene expressions of apoptosis-inducing factor were significantly (*p* < 0.05) upregulated by SHAM treatment, especially LOC107831784 (log_2_FC = 1.37) and LOC107827516 (log_2_FC = 1.76). In addition, the qRT-PCR analysis also showed that these genes were upregulated on SHAM treatment (Figure 9B), indicating that the impairment of the tobacco root development was associated with cell apoptosis induced by AOX inhibition.

### Methyl Jasmonate Cannot Restore Root Development Defects Mediated by AOX Inhibition

**Figure 10:**
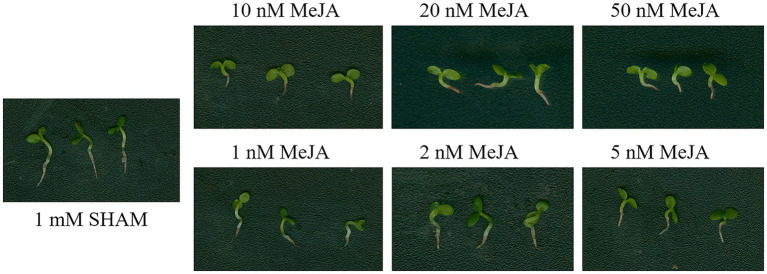
Effects of adding methyl jasmonate (MeJA) on the root development of SHAM treatment. The root development on the 8th day of samples treated with 1 mM SHAM (CK) and treated with SHAM plus different concentrations of MeJA are shown.

Considering that SHAM was reported as an inhibitor of lipoxygenase, which is a key enzyme of jasmonate (JA) biosynthesis, the DEGs related to lipoxygenase were analyzed. As shown in Supplementary Table S2, five DEGs related to lipoxygenase were regulated by SHAM. Of these, three genes, namely, LOC107800317, LOC107760301, and LOC107763327, were downregulated, while LOC107803018 and LOC107770253 were upregulated by SHAM treatment. In order to study whether MeJA helps to reduce the inhibitory effect of SHAM, the different concentrations of MeJA were introduced into the media containing 1 mM SHAM. The results showed that 100–500 nM MeJA did not restore the root development defects mediated by AOX inhibition, but aggravated the root inhibition (data not shown). Subsequently, we reduced the concentrations of MeJA to 10–50 nM and observed the same results, i.e., there were some cumulative damages to the root development (Figure 10). When the applied concentrations of MeJA were decreased to 1–5 nM, no restoration effects were still occurred (Figure 10). These results indicate that MeJA cannot restore root development defects mediated by AOX inhibition. It should be noted that, furthermore, the gene expression of *TIFY 6B* [also known as JA ZIM-domain (JAZ3) protein] was significantly downregulated (log_2_FC = −4.75) by SHAM treatment (Supplementary Table S2).

### Auxin and ROS Scavenger Can Partially Restore Root Development Defects

**Figure 11:**
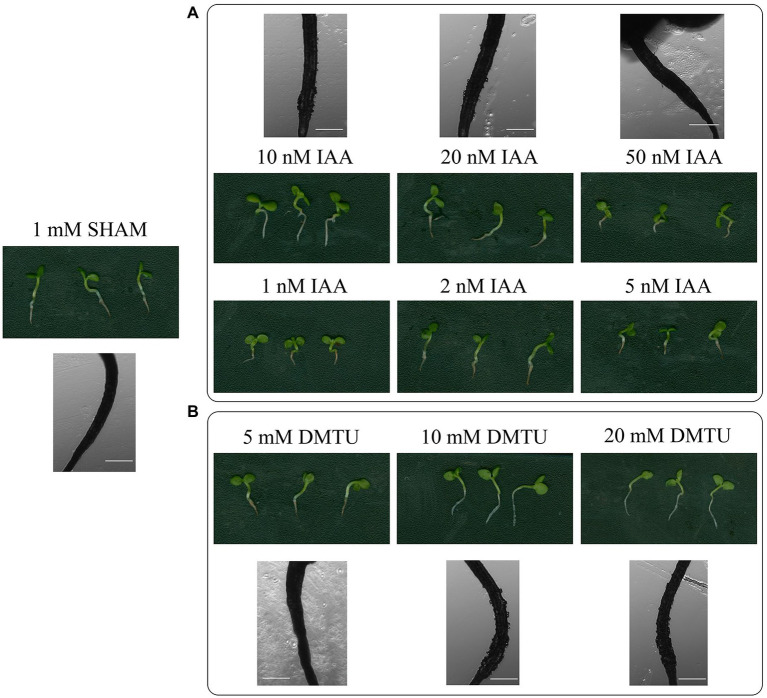
Effects of adding indoleacetic acid (IAA) and dimethylthiourea (DMTU) on the root development of SHAM treatment. The root development on the 8th day of samples treated with 1 mM SHAM and treated with SHAM plus different concentrations of IAA **(A)** and DMTU **(B)** are shown. In addition, the representative root hairs were observed with a microscope. Scale bar, 500 μm.

Given that auxin biosynthesis and ROS overaccumulated in the SHAM-treated roots, different concentrations of IAA and ROS scavenger (DMTU) were introduced into the media. As shown in Figure 11, when 50–100 nM IAA were added to the media containing 1 mM SHAM, the development of these roots was further inhibited compared with roots treated with 1 mM SHAM. When the concentration of IAA reduced to 10–20 nM, some restoration effects were occurred, e.g., the root length was longer, and a small amount of root hairs appeared (very short) in the zone of differentiation than the 1 mM SHAM-treated roots (Figure 11A). However, there was no significant difference from the roots treated with 1 mM SHAM when the concentration of IAA reduced to 1–5 nM (Figure 11A).

When DMTU was introduced into the media containing 1 mM SHAM, different effects were observed. It should be noted that 5 mM DMTU did not change the root inhibitory effect caused by 1 mM SHAM. When 10 mM DMTU was used, a small amount of root hairs (very short) appeared in the zone of differentiation. When the concentration was further increased to 20 mM DMTU, the restoration effect was not further improved (Figure 11B). These results indicate that the inhibition of auxin and the excessive accumulation of ROS are only part of the reasons why AOX inhibition mediated the hindrance of root development.

## Discussion

Plant root is an important organ for plant growth and development. Root development and root hair formation are the complexities regulated by plant hormones such as auxin. Although there have been many reports on the regulation of root development and root hair formation, there is still a large number of unknowns that need to be explored. In this study, we found that the inhibition of AOX with different concentrations of SHAM affected the tobacco root development, especially the root hair growth. The higher concentrations of SHAM were applied, and the more severe repressions and damages on the main root and root hair development were observed.

It has been shown that ROS such as superoxide and hydrogen peroxide is involved in controlling root growth and root hair formation (Foreman et al., 2003; Tsukagoshi, 2016). One of the important roles of ROS in root development is their function in modifying cell walls (O’Brien et al., 2012; Karkonen and Kuchitsu, 2015). ROS also plays a role in determining the size of the meristematic zone by regulating the cell cycle (Tsukagoshi, 2012, 2016). However, it is of interest to note that AOX inhibition with SHAM inhibited the root architecture and more H_2_O_2_ contents and cell deaths were observed than that of the control (Figures 1, 2), indicating that oxidative damage occurred when SHAM was applied. Combined with the earlier reports, we hypothesized that ROS plays a dual role in root development. That is, the lower concentration of ROS contributes to root development, but the higher levels of ROS cause oxidative damage and apoptosis as found in this study.

The relationship between ROS and AOX has been concerned and studied for many years. AOX has been demonstrated to play important roles in reducing ROS accumulation, especially when plants were subjected to biotic and abiotic stresses (Cvetkovska and Vanlerberghe, 2013). Conversely, a lack of AOX leads to ROS burst in plant cells and compromises the capacity to recover from environmental stress (Wang and Vanlerberghe, 2013; Saha et al., 2016; Selinski et al., 2018). Therefore, it is reasonable that the observed oxidative damage was associated with AOX inhibition.

To further investigated that whether AOX inhibition affected other key factors that related to root development and root hair formation, RNA-Seq was performed, and in this study, we found that more than 5,000 genes were significantly regulated by AOX inhibitor SHAM (Figure 3). Interestingly, a large amount of DEGs was related to ROS scavenging system. In comparison, the gene expressions of SOD showed upregulation on SHAM treatment, while most of the gene expressions of H_2_O_2_ scavenging enzymes were downregulated by SHAM, which is consistent with the results of ROS assay i.e., less superoxide ion but more H_2_O_2_ accumulated in the SHAM-treated roots than that in the control (Figure 2). In addition, many genes related to GSTs were upregulated after SHAM treatment, which further indicates the severe oxidative damage that occurred in the root cells, because GSTs are notable for their role in detoxification reactions in plants (Kumar and Trivedi, 2018). GSTs are also known to catalyze glutathione (GSH) conjugation to electrophilic compounds, and parts of them possess GPX activities playing a crucial role in plant antioxidative defense (Edwards et al., 2000). However, the increase in the GST and GPX gene expressions in this study indicates to a greater extent that the root development was blocked and damaged by AOX inhibition, rather than effectively alleviated these damages. Likewise, the expressions of PAO and PPO genes were downregulated by AOX inhibition, which further suggests that oxidative damages were severe in SHAM-treated tobacco roots. It has been demonstrated that PAO and PPO play crucial roles in plant development and abiotic stress tolerance, by triggering the production of H_2_O_2_ and toxic quinone (Alcazar et al., 2010; Taranto et al., 2017; Yu et al., 2019). Obviously, the reduction in PAO and PPO gene expressions on SHAM treatment is to reduce further oxidative damage as much as possible caused by AOX inhibition.

In this study, exogenous application of ROS scavenger (DMTU) did not significantly rescue the root development defects caused by AOX inhibition, although some short root hairs appeared in the zone of differentiation after adding very high DMTU (10–20 mM) to the medium. In fact, the exogenous application of DMTU could not completely eliminate the oxidative damage caused by AOX inhibition in the roots. To explain this, there should be at least two reasons: on the one hand, the production rate of ROS (caused by AOX inhibition) and the clearance rate of ROS (DMTU) may be different, with the former being faster. On the other hand, ROS scavenging enzyme was significantly downregulated by AOX inhibition as mentioned above. The precise balance of ROS plays an important role in regulating plant growth and development (including root and root hair formation). Previous study has shown that if the homeostasis of ROS in plants is altered, it will lead to changes in root growth and development (Livanos et al., 2012; Sundaravelpandian et al., 2013; Tsukagoshi, 2016).

Changes in cell walls are also important for root growth and root hair formation. In general, cell wall loosening is involved in root cell elongation and root hair initiation (Tominaga-Wada et al., 2011; Petricka et al., 2012). Notably, this study showed that AOX inhibition affected the cell wall-related gene expression and most of them showed downregulation on SHAM treatment (Table 1). Of these, it should be noted that the gene expressions of xyloglucan endotransglucosylase and pectinesterase were significantly downregulated by SHAM treatment. Xyloglucan endotransglucosylases are enzymes that cleave or catalyze the transfer of xyloglucan chains, which are cell wall polysaccharides (Vissenberg et al., 2003; Tominaga-Wada et al., 2011). Root hair initiation is primarily coupled to a highly localized increase in xyloglucan endotransglucosylase action at the basal initiation site of hair cells, followed by general distribution over the surface of the growing root hair (Vissenberg et al., 2001; Tominaga-Wada et al., 2011). Importantly, xyloglucan endotransglucosylase action takes place at the site of future bulge formation, where the root hair cell locally loosens its cell wall structure, suggesting important roles for xyloglucan endotransglucosylase at the beginning of root hair tip growth (Vissenberg et al., 2001). In addition to xyloglucan endotransglucosylase, pectinesterase (also called pectylhydrolase and pectinmethylesterase) plays a role in the modulation of plant cell wall extension during pollen germination and pollen tube growth, abscission, stem elongation, tuber yield, and root development (Palin and Geitmann, 2012; Wu et al., 2018). It has been demonstrated that pectinesterase action in stimulating the activity of cell wall hydrolases contributing to cell wall loosening (Nari et al., 1991; Giovane et al., 2004). The study from Wen et al. (1999) stated that partial inhibition of the gene expression of pectinesterase by antisense mRNA in transgenic pea hairy roots prevented the normal separation of root border cells from the root tip into the external environment and showed reduced root elongation. Taken together, the downregulation of xyloglucan endotransglucosylase and pectinesterase gene expressions after SHAM treatment should be one of the main factors affecting the root elongation and root hair formation observed in this study.

Other important factors affecting the root development and root hair formation are plant hormones, where the role of auxin is particularly crucial. Auxin is a well-characterized hormone that influences many plant developmental processes and acts as a positive regulator of root hair development (Vissenberg et al., 2020). In citrus, a positive correlation was observed between the endogenous auxin level and root-hair number in the root hair zone (Zhang et al., 2018). In this study, we found that the amount of gene expression associated with auxin syntheses (*TAA1* and *YUCs*) and the response was significantly downregulated by AOX inhibitor SHAM (Figure 7), suggesting that the effect of AOX inhibition on root development involves auxin synthesis. It is notable that the gene expression of auxin receptor such as *TIR1* showed downregulation, while the negative regulator of auxin signaling pathway such as *AUX/IAA* genes showed upregulation after SHAM treatment (Figure 7), indicating that auxin signaling transduction was also suppressed along with AOX inhibition.

It seems that AOX inhibition also affected the auxin homeostasis based on the results in this study. For example, the results showed that the expression of *GH3* family genes was upregulated following treatment with SHAM (Figure 7). It is known that GH3, which encodes auxin-conjugating enzymes, functions in plant growth and development by the regulation of IAA homeostasis (Park et al., 2007). GH3 promotes the inactivation of IAA by amide conjugates with Asp, Glu, Ala, Gly, Val, and Leu (Staswick et al., 2005; Aoi et al., 2020). Importantly, evidence has demonstrated that the overproduction of an IAA-conjugating GH3 enzyme exhibited auxin-deficient traits, including reduced growth, altered leaf shape, and root meristem size (Park et al., 2007; Di Mambro et al., 2017). Together, it appears that the regulated expression of *GH3* family genes may be involved in the repression of auxin levels by AOX inhibition.

In this study, we found that the gene expression of lipoxygenase, a key enzyme of JA biosynthesis, was downregulated (log_2_FC = −1.26) by SHAM treatment. Previous study has shown that SHAM may be involved in the regulation of root development by inhibiting JA biosynthesis (Zhu et al., 2006). However, no restoration effect was observed when the different concentrations of MeJA were introduced into the media containing 1 mM SHAM. Conversely, the root development inhibition was aggravated when ≥10 nM MeJA was added into the media containing 1 mM SHAM. In fact, our research found that although SHAM inhibited the expression of some *LOX* genes, it also more significantly inhibited the expression of *JAZ3* gene (log_2_FC = −4.75), which is a negative regulator in the JA signal transduction pathway (Pauwels and Goossens, 2011). It should be noted that, unlike the replenishment effect of MeJA, the exogenous supplement of IAA conferred partial restoration effects to the SHAM-treated roots. In this study, we found that when 10–20 nM IAA were added into the media, tobacco roots were longer and some short root hairs emerged in the zone of differentiation compared with the SHAM-treated roots. Higher IAA concentrations had a damaging effect, while lower IAA had no restorative effect on the tobacco roots treated with 1 mM SHAM. Based on these results, we cannot rule out the role of JA in root development, but certainly, these results showed that auxin must be involved in the influence of AOX pathway inhibition on the tobacco root development. In addition, the recovery effects of auxin were limited, which also indicate that there should be other factors involved in root development defects mediated by AOX inhibition. Although ethylene has been proposed to play a synergistically role in root development with auxin, no significant downregulation of gene expression related to ethylene synthesis and signaling transduction pathway occurred in the AOX-inhibited samples in this study (Figure 8). It was shown that ethylene is a positive regulator for root development and root hair formation (Tanimoto et al., 1995; Rahman et al., 2002). Ethylene biosynthesis inhibitor aminoethoxyvinylglycine (AVG) and silver ion (i.e., an inhibitor of ethylene perception) have been found to inhibit root hair formation (Tanimoto et al., 1995). In addition, evidence is provided that ethylene modulates stem cell division in the root system (Ortega-Martínez et al., 2007). Notably, our study showed that the gene expression of ACO, a key enzyme of ethylene biosynthesis, was upregulated on AOX inhibition. The same expression changes also included *EIN3* and *ERF1* genes, indicating that the AOX inhibition caused defects in the root development are not through the interference of the ethylene pathway. On the contrary, the induction of ethylene synthesis and perception in this study might be due to the oxidative damage caused by AOX inhibition.

It is worth mentioning that the gene expression of LOC107787646, which encodes acetate/butyrate-CoA ligase, was significantly downregulated by SHAM treatment (Figure 5). Previous studies have shown that acetate/butyrate-CoA ligase is probably involved in the activation of exogenous acetate for entry into the glyoxylate cycle and plays a role to prevent carbon loss from peroxisomes during lipid mobilization (Turner et al., 2005; Hooks et al., 2010). It has been suggested that the levels of acetyl-CoA are critical for normal seedling development (Oliver et al., 2009). Therefore, it is reasonable to speculate that the AOX inhibition that affects the root development is involved in the feedback regulation of lipid metabolism and subsequent energy conversion. Nevertheless, a lot of studies have to be done to analyze the relationship between AOX activity, lipid metabolism, and acetyl-CoA pools.

## Conclusion

In this study, we found that the inhibition of AOX impaired the root development and root hair formation. It seems that the inhibition of AOX disrupted the homeostasis of mitochondrial respiration by reducing total respiration and then caused ROS bursts that damaged the root cellular metabolism. The biochemical determination and the transcriptome analysis showed that AOX inhibition was involved in the regulation of a large number of gene expressions related to the auxin biosynthesis and perception, as well as the ROS scavenging system, cell cycle, RMGF1, and cell wall metabolism, indicating that the AOX pathway plays important roles in the tobacco root architecture. However, more research is needed in the future to reveal the effects and mechanisms of AOX in root development.

## Data Availability Statement

The data presented in the study are deposited in the sequence read archive (SRA) repository, accession numbers (SRR13717005–SRR13717010).

## Author Contributions

FX conceived the project and supervised this study. L-LY and FX wrote the manuscript. L-LY, YL, and YP performed the experiments. L-LY, YL, X-XG, and FX contributed to the data analysis. All authors contributed to the article and approved the submitted version.

### Conflict of Interest

The authors declare that the research was conducted in the absence of any commercial or financial relationships that could be construed as a potential conflict of interest.

## Footnotes

^1^http://www.genome.jp/kegg/
