# Supplementary material for: Alternative Oxidase Inhibition Impairs Tobacco Root Development and Root Hair Formation
Source: Front Plant Sci. 2021 Jun 24;12:664792. doi: 10.3389/fpls.2021.664792 (PMC8264555; doi:10.3389/fpls.2021.664792)
Supplement: Supplementary file 1 [file Data_Sheet_1.docx]

***Supplementary Material***

**1. Supplementary table**

**TABLE S1 Primers used for qRT-PCR**

| **Gene ID** | | **Forward 5’→3’** | **Reverse 5’→3’** | **note** |
| --- | --- | --- | --- | --- |
| 1 | LOC107825840 | TTTCTTTGCTCGTAGATGTG | TTGTAGGGTACTCAGGGTAGT | Random selected genes |
| 2 | LOC107790589 | GTCGGCGATGAGATGGAG | TCGGAAAGAGCACTGAGAAG |  |
| 3 | LOC107784906 | CAGCAATAGCCTCCCTCT | CGAAGCACATCACCGACT |  |
| 4 | LOC107821957 | ATGTATGCCTCGTCTGAATG | AGGGTGATGCTCCTGTGC |  |
| 5 | LOC107762104 | TCCGAGGAGGGACAAGTG | GGCAATAAAACGGCAAGG |  |
| 6 | LOC107786140 | CGATGATGTTTGTCTCCC | GCGTGGCTATGATTTGTAC |  |
| 7 | LOC107770509 | TCAGTCAGGTTGACATAGCGTA | CGACCACTTTCTGGGGAT |  |
| 8 | LOC107794811 | GAGTCGTTGCTACCCTCA | TTGGTTTCCTTTGTCTGC |  |
| 9 | LOC107781126 | CAGACGGGACTCGGATGT | CCTTGGCTCTTTGGCTTG |  |
| 10 | LOC107831784 | TCCCTCATTGCCAAGTCC | GGCTCCACCATTGCTCTT | Apoptosis related genes |
| 11 | LOC107827516 | GATGAGTGGAGGAAAGGA | TAACAAGCCAAGGTAAGG |  |
| 12 | LOC107793422 | ATTCAGAAGTTGGTGGTT | CTATGCCTGTTTGTCCTC |  |

**TABLE S2 DEGs related to lipoxygenase**

| Gene id | log_2_FC | p-value | Gene description | TF family |
| --- | --- | --- | --- | --- |
| 107800317 | -1.26 | 4.72E-23 | linoleate 13S-lipoxygenase 2-1%2C chloroplastic-like | Lipoxygenase |
| 107803018 | 1.18 | 1.35E-11 | probable linoleate 9S-lipoxygenase 5%2C transcript variant X2 | Lipoxygenase |
| 107770253 | 1.02 | 0.0001 | probable linoleate 9S-lipoxygenase 5%2C transcript variant X4 | Lipoxygenase |
| 107763327 | -3.97 | 0.0034 | probable linoleate 9S-lipoxygenase 5 | Lipoxygenase |
| 107807057 | -4.75 | 0.0020 | protein TIFY 6B-like, JAZ | Zim |

**2. Supplementary Figure**

**
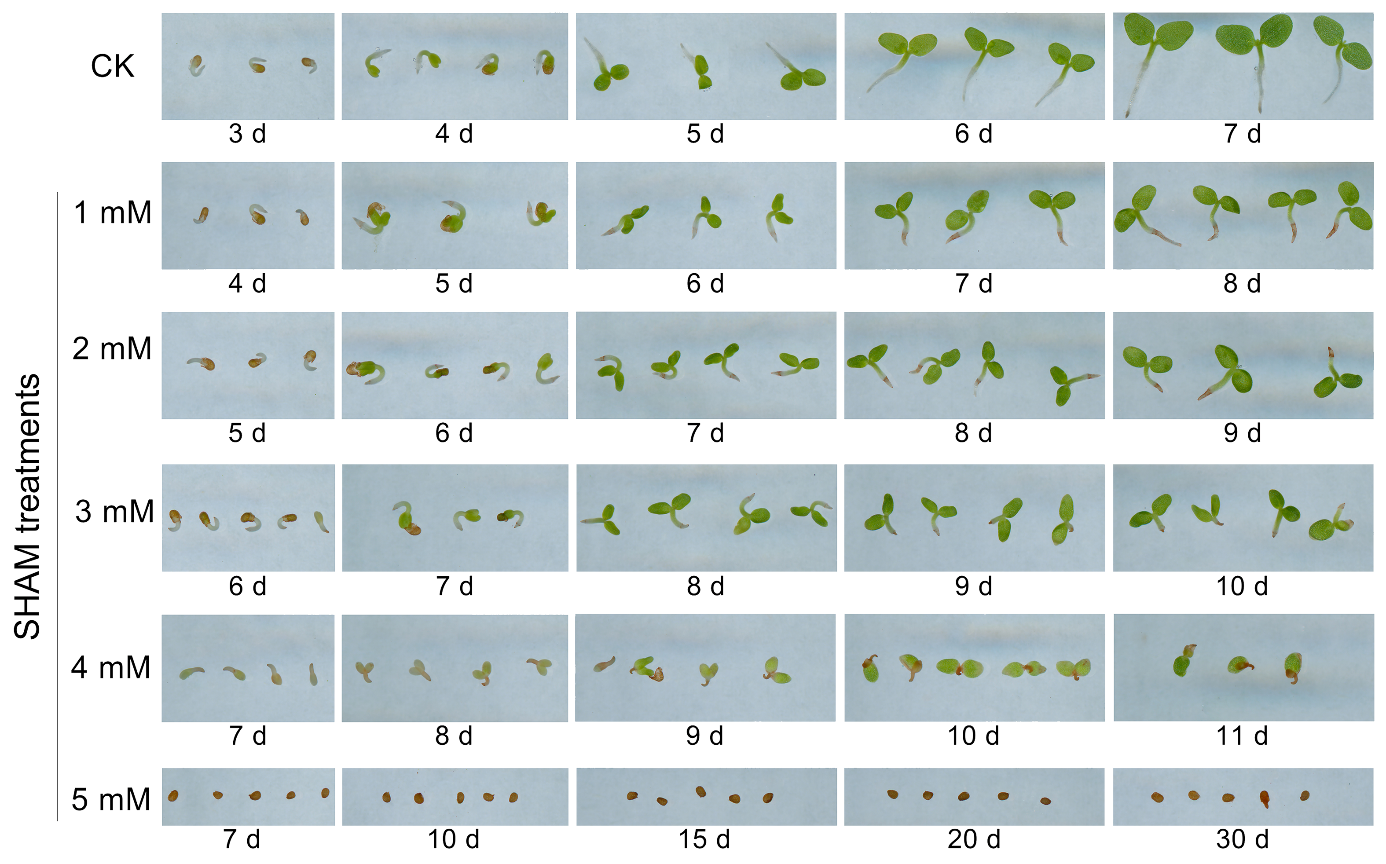
**

**FIGURE S1.** **Effects of different concentrations of SHAM treatment on root development of tobacco**. Roots development under normal condition (CK) and under different concentrations of SHAM treatment are shown. For SHAM treatments, the seeds were germinated in 1/2 MS containing 1 mM, 2 mM, 3 mM, 4 mM and 5 mM SHAM, respectively, under 16h-light/8h-dark photoperiod, 70% relative humanity.


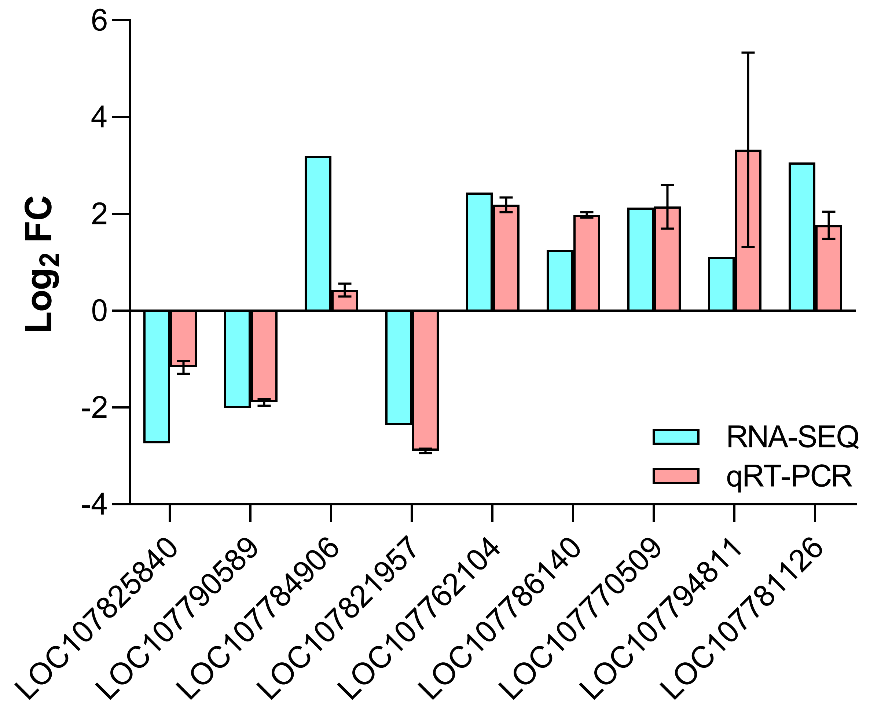


**FIGURE S2.** **Quantitative analysis of some differentially expressed genes by RNA-SEQ and qRT-PCR.** Data are presented as log_2_FC values in comparison with the control. FC, fold change.
